# Elevated Serum Aspergillus Fumigatus-Specific Immunoglobulin G in Type 2 Chronic Rhinosinusitis

**DOI:** 10.7150/ijms.83327

**Published:** 2023-05-12

**Authors:** Yun-Chen Chang, Ta-Jen Lee, Chi-Che Huang, Po-Hung Chang, Yi-Wei Chen, Chia-Hsiang Fu

**Affiliations:** 1 Department of Otolaryngology-Head and Neck Surgery, Linkou Chang Gung Memorial Hospital, No. 5 Fu-Shin Street, Guishan District, Taoyuan City, 333, Taiwan.; 2 Graduate Institute of Clinical Medical Sciences, College of Medicine, Chang Gung University, No. 259, Wenhua 1st Rd., Guishan Dist., Taoyuan City 333, Taiwan.

**Keywords:** *Aspergillus fumigatus*-specific immunoglobulin G, endotype, phenotype, primary chronic rhinosinusitis, type 2

## Abstract

**Background:** *Aspergillus fumigatus*-specific immunoglobulin G (Af-sIgG) has been applied to diagnose allergic bronchopulmonary aspergillosis, a hypersensitivity reaction to the colonization of the fungus in the lower airways. In the upper airways, it has been reported to be involved in allergic fungal rhinosinusitis and local fungal rhinosinusitis. However, in primary chronic rhinosinusitis (CRS), a more common upper airway disease, the role of Af-sIgG remains unclear.

**Objective:** The aim of our study was to investigate the role of serum Af-sIgG levels in primary CRS patients.

**Methods:** We prospectively recruited patients diagnosed with bilateral primary CRS and patients with nasal septal deviation as the non-CRS group. Patients in the primary CRS group were further classified into two endotypes, including type 2 (T2) and non-T2 groups. Serum samples collected were sent for Af-sIgG analysis. Potential factors and surgical outcomes were analyzed.

**Results:** Forty-eight patients with a diagnosis of primary CRS (including 28 with T2 and 20 with non-T2 CRS) and 22 patients in the non-CRS group were recruited. The T2 CRS group had significantly higher serum Af-sIgG levels than the non-T2 CRS group (odds ratio 10.2 with Af-sIgG more than 27.6 mg/L; *p* < 0.001). Further multivariate logistic regression showed that the serum Af-sIgG level was the independent factor for early disease recurrence within one year in primary CRS patients. The optimal cutoff value of the serum Af-sIgG level to predict postoperative recurrence was 27.1 mg/L (odds ratio 15.1, *p* = 0.013).

**Conclusions:** We suggest that the serum Af-sIgG level is a practical marker to detect T2 inflammation and the surgical outcome of primary CRS. By applying this feasible test, we may be able to achieve optimal treatment for every individual with primary CRS. This study may provide physicians with a reference for future clinical applications in dealing with primary CRS.

## Introduction

The human respiratory tract is frequently exposed to airborne fungi and fungal enzymes. Aspergillus fungi species are common and can affect both the upper and the lower respiratory tracts. In the lower airways, the clinical spectrum of hypersensitivity manifestations includes *Aspergillus fumigatus* (Af)-induced asthma and allergic bronchopulmonary aspergillosis (ABPA) 1, 2. ABPA, predominantly a disease of asthmatics, has been well established worldwide for nearly six decades, with reports from around the globe. Katzenstein *et al.* retrospectively reviewed 119 surgically obtained specimens from the paranasal sinuses and found that seven patients had pathologic findings characteristic of mucoid impaction of ABPA 3. Currently, the detection of *Aspergillus fumigatus*-specific immunoglobulin G (Af-sIgG) antibodies against lower airway diseases has been documented to be useful in diagnosis, and a positive Af-sIgG level indicates more active disease and worse outcomes of ABPA 4.

The concept of united airway disease has been proposed as a pathological continuum due to the interactions between the upper and lower airways in terms of atopic, infectious, and inflammatory links 5. The nose and lower respiratory tract share similar physiological and histological properties 6, 7. Aspergillus has a high capacity to colonize the bronchial tract of asthmatic patients, causing severe persistent asthma and lower lung function, sometimes leading to ABPA 8. The role of Aspergillus in upper airway diseases has been established, such as colonizing the sinus mucosa and producing a dense conglomeration of hyphae in sinus cavities causing fungal rhinosinusitis (a fungal ball) and triggering the Th2 lymphocyte response with eosinophilic mucin containing fungal hyphae in the sinus, which is the hallmark of allergic fungal rhinosinusitis (AFRS) 9.

To the best of our knowledge, no research in the English literature has studied the relationship between Af-sIgG and primary chronic rhinosinusitis (CRS). Although immunologic reactions to fungi are speculated to be one of the causes of CRS 10, 11, the ability to visually detect the presence of fungal organisms is challenging.

A previous study revealed that one or more species of fungi could be cultured from mucin in 96% of CRS patients and 100% of healthy controls 10, 12. In contrast, histopathologic identification of these organisms using the Grocott methanamine silver stain (GMS) revealed the presence of fungal elements within the mucin in 81% of patients 10, 12. However, the connection between the appearance of serum fungus-specific antibodies and disease severity or outcome has yet to be established.

CRS is a complex, multifactorial airway disease. Primary CRS is defined when the disorder is limited to the airway or respiratory system only 13. In the European Position Paper on Rhinosinusitis and Nasal Polyps 2020 (EPOS 2020), primary CRS can be considered type 2 (T2) and non-T2 according to the cytokines involved 14. T2 CRS, characterized by the activation of type 2 helper T cells and type 2 innate lymphocyte cells, which produce the key cytokines interleukin (IL)-4, IL-5, and IL-13, is a more severe disease and has a higher postoperative recurrence rate 13, 15. The recurrence rate of T2 CRS after surgery was 8%-45% in a previous study 16. We assumed that Aspergillus colonization plays a role in T2 CRS, causing more severe disease and worse outcomes, similar to the characteristics of ABPA mentioned above. Among all Aspergillus species, Af has been stated to be the most common type found in CRS 17. Thus, the aim of our study was to investigate the role of serum Af-sIgG levels in distinct types of primary CRS. Furthermore, the correlation between the serum Af-sIgG levels and disease entity, disease severity, and treatment response was analyzed.

## Methods

### Patient population

We prospectively recruited patients diagnosed with bilateral primary CRS refractory to at least three months of medical treatment who underwent bilateral functional endoscopic sinus surgery (FESS) by the same physician. Patients with nasal septal deviation without CRS confirmed by transnasal endoscopy were included as the control group. Patients with secondary CRS, sinonasal neoplasms, previous sinonasal surgery or radiotherapy, age less than 20 years old, or current pregnancy were excluded. To evaluate the feasibility of Af-sIgG in common primary CRS, patients who met the criteria of AFRS were excluded, although it is extremely rare in Taiwan 18, 19. All enrolled patients met the diagnostic criteria of CRS in EPOS 2020 14. The clinical diagnosis of CRS was made by transnasal endoscopic findings or computed tomography (CT) scans in the outpatient department. The enrolled CRS patients completed the postoperative regimen and regular follow-up for at least one year. None of the patients received antibiotics, corticosteroids, or antihistamines within 2 weeks prior to enrollment.

### Measurement of serum Af-sIgG levels

The serum samples collected from the enrolled patients one day before surgery were sent for Af-sIgG analysis with a commercial ImmunoCAP system (Phadia®, ThermoFisher Scientific, MA, USA) using automated fluorescent enzyme immunoassay technology. Anti-IgG antibody was covalently bound to ImmunoCAP and reacted with Af-sIgG in the patient's serum after being added to the sample. The enzyme-bound antibody forms a complex with the Af-sIgG antibody. Following incubation for a period, the unbound enzyme-anti-IgG antibody is washed away, and the bound complex is then incubated with a developing agent. After the reaction is completed, the fluorescence of the eluate is measured. By measuring the fluorescence response of the patient sample, the correct response concentration can be obtained by comparison with the calibration curve.

### CRS classification

According to the cytokines involved in disease development, primary CRS was also classified into endotypes, including T2 and non-T2 CRS. According to EPOS 2020 and a previous study, T2 CRS was defined as a blood eosinophil count > 250 cells/µL or met at least two of the following four criteria: blood eosinophils > 150 cells/µL, total IgE > 100 KU/L, allergic sensitization and comorbid late-onset asthma 14, 20. CRS patients were also classified into two groups based on their phenotypes: those with nasal polyps (CRSwNP) and those without polyps (CRSsNP). The existence of nasal polyps was based on endoscopic findings or pathology reports or both.

### Surgical treatment and postoperative regimen

Endoscopic sinus surgery was performed for CRS management, applying a bidirectional method that combines anterior-to-posterior and posterior-to-anterior approaches 21. After surgery, all patients were scheduled for regular follow-ups at our outpatient department as previously described 22. Briefly, patients were treated with Augmentin^®^ 30 mg/kg/day for 1 week postoperatively, and at 2 weeks after surgery, nasal corticosteroid spray (mometasone furoate, 200 μg/day) and nasal saline irrigation (250 ml once daily) were initiated for 3 months. Postoperative visits were performed weekly for 1 month, monthly for 6 months, and every 3 months for at least another 6 months.

Recurrence of CRS was defined as a relapse of purulent sinus discharge or recurrence of nasal polyps under endoscopy for at least 2 months despite a rescue regimen of antibiotics, oral steroids, corticosteroid nasal spray, or intensive nasal irrigation during postoperative follow-up 22. A mild polypoid appearance of the sinus or turbinate mucosa would not be considered a recurrence.

### Subjective and objective measurements

All patients completed Sino-Nasal Outcome Test-22 (SNOT-22) questionnaires one day before surgery 23. The SNOT-22 tool is a validated instrument developed to quantify self-reported measures of symptom severity associated with sinonasal conditions. The SNOT-22 tool covers various symptoms, physical problems, functional limitations, and emotional consequences of having a sinonasal disorder. The item scores range from 0 to 5, with higher scores indicating more severe symptoms 24.

Primary CRS patients underwent nasal endoscopy examination and sinus CT scans to confirm the diagnosis before surgical treatment. The Lund-Kennedy (LK) endoscopy score was recorded at the outpatient department 25, and preoperative CT scans were evaluated by a senior surgeon in a blinded fashion to record the sinusitis severity using the Lund-Mackay (LM) system 26.

### Statistical analysis

Statistical analysis was performed using MedCalc and GraphPad Prism version 5.0. The Mann‒Whitney U test was used to compare significant differences between groups. A receiver operating characteristic (ROC) curve was used to determine the cutoff limit of serum Af-sIgG in the derivation cohort. The best cutoff value was described using Youden's index. Numerical data were analyzed with the Kruskal‒Wallis test between groups and are presented as the median with 95% confidence interval (CI). Categorical data were analyzed with the chi-square test and Fisher's exact test and are presented as percentages. In the multivariate logistic regression (MLR) analysis, backward stepwise logistic regression was used for the evaluation of independent factors for recurrence of CRS, and the coefficient and odds with 95% confidence interval are presented. A value of *p* < 0.05 was considered statistically significant.

Patients who had disease recurrence or not were compared using univariate analysis (the chi-square test and Student's t test were used for nominal and numerical variables, respectively). Variables with a *p* value < 0.25 were put into the subsequent backward stepwise logistic regression to evaluate independent factors for disease recurrence 27. The performance of the MLR analysis was assessed by determining its discrimination and calibration. The discrimination was measured by calculating the area under the receiver operating characteristic curve (AUROC). The calibration was assessed using the Hosmer-Lemeshow test Ĉ-test (with *p* > 0.05 indicating no significant difference between the predicted and observed outcomes) 28.

## Results

### Study population

Eventually, 70 patients who completed the 1-year postoperative follow-up were recruited, including 48 patients with a diagnosis of CRS (28 with T2 CRS, 20 with non-T2 CRS) and 22 patients in the non-CRS group. One patient with AFRS was excluded from enrollment. Comparing patients in the T2 CRS group with those in the non-T2 groups, patients in the T2 CRS group had a significantly higher incidence of comorbid asthma history, allergy status, serum eosinophil count, serum total IgE level, SNOT-22 score and serum Af-sIgG level than non-T2 CRS patients. In addition, as expected, the T2 CRS group had higher LK endoscopy and LM CT scores than non-T2 CRS patients, although the differences did not reach statistical significance. There are no particularly conspicuous data in the non-CRS group, our healthy control group. Detailed demographics of each group are shown in **Table 1**.

### Serum Af-sIgG levels in the primary CRS groups by endotype and phenotype

**Figure 1:**
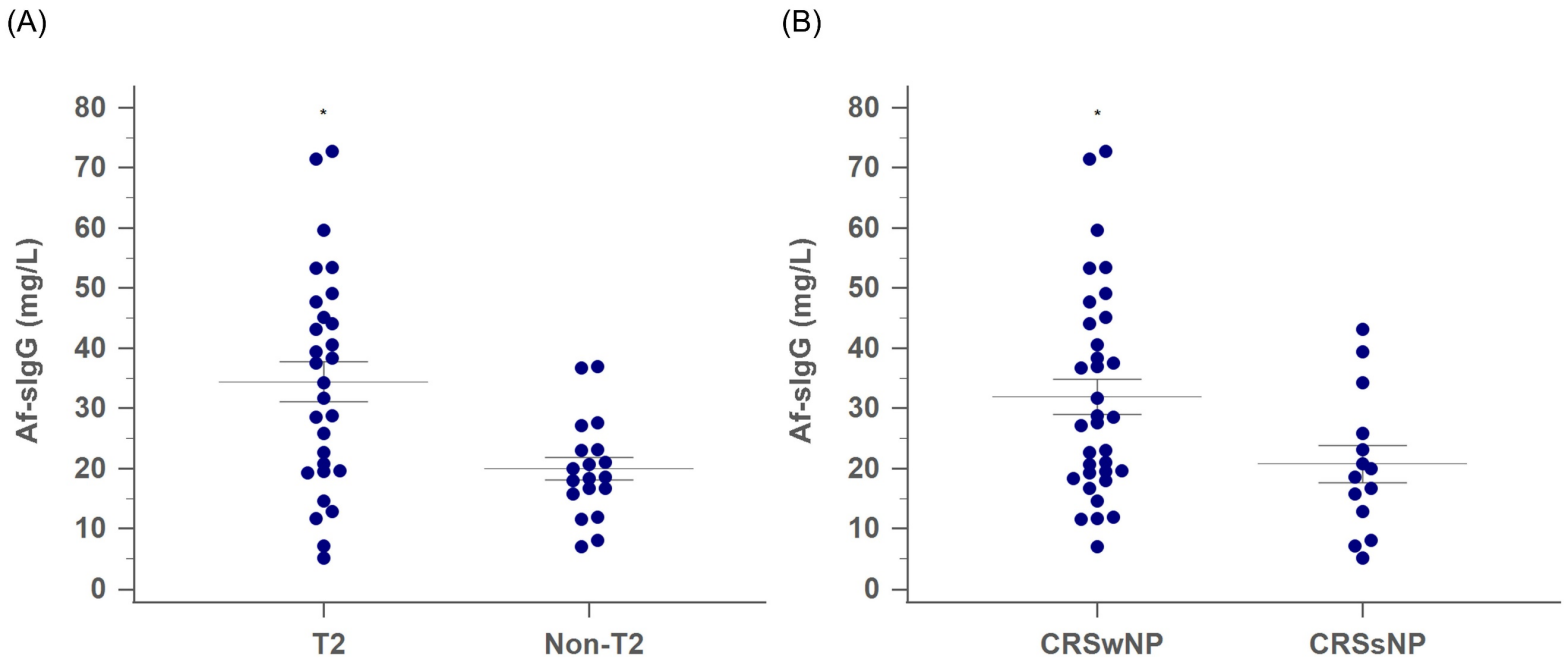
The comparison of serum Af-sIgG level (mg/L, mean ± SEM) between (A) T2 CRS and non-T2 CRS groups, and (B) CRSwNP and CRSsNP groups. Internal horizontal lines showing the standard deviation of the mean. Abbreviations: Af-sIgG, Aspergillus fumigatus-specific immunoglobulin G; T2, type 2; CRS, chronic rhinosinusitis; CRSwNP, chronic rhinosinusitis with nasal polyps; CRSsNP, chronic rhinosinusitis without nasal polyps; *p < 0.05.

Among the 48 patients with primary CRS, the T2 group had significantly higher serum Af-sIgG levels than the non-T2 group (35.9 mg/L and 19.1 mg/L for the T2 and non-T2 groups, respectively; *p* = 0.003, **Figure 1A**). Furthermore, while CRS patients were categorized according to the existence of nasal polyps, there were 34 patients in the CRSwNP group and 14 patients in the CRSsNP group. The serum Af-sIgG level in the CRSwNP group was markedly higher than that in the CRSsNP group (32.0 mg/L and 20.8 mg/L for the CRSwNP and CRSsNP groups, respectively; *p* = 0.038, **Figure 1B**).

**Figure 2:**
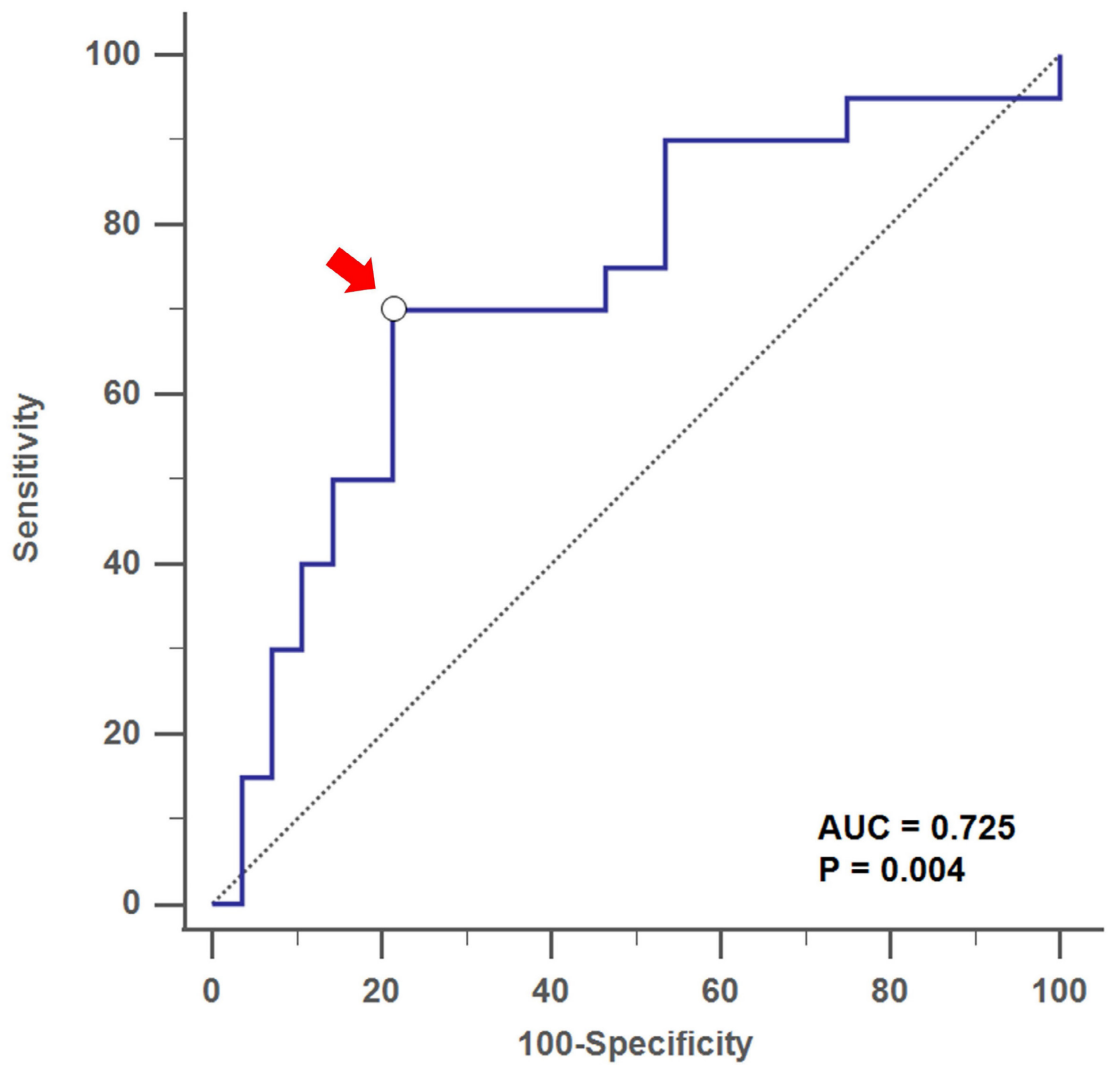
Receiver operating characteristic (ROC) curve analysis showed the optimal cutoff value of serum Af-sIgG level (arrow) at 27.6 mg/L to predict T2 CRS, with AUC = 0.725 (*p* = 0.004) Abbreviations: Af-sIgG, Aspergillus fumigatus-specific immunoglobulin G; T2, type 2; CRS, chronic rhinosinusitis; AUC = area under the curve.

By using ROC curves and Youden's index, we found that the optimal cutoff value of the serum Af-sIgG level to predict T2 CRS was 27.6 mg/L, with a sensitivity of 70.0%, a specificity of 78.6%, a positive predictive value (PPV) of 70.1% and a negative predictive value (NPV) of 78.6% (area under curve, AUC = 0.725, *p* = 0.004; **Figure 2**). For patients with primary CRS who had serum Af-IgG levels above 27.6 mg/L, there was a 10.2-fold increased probability of being categorized as a T2 response (*p* < 0.001 by logistic regression).

### Serum Af-specific IgG levels and postoperative recurrence

Patients with primary CRS followed the postoperative regimen regularly for at least 12 months after surgery. Nine patients had disease recurrence during regular follow-up. The background, demographic and laboratory data were compared between the recurrence and nonrecurrence groups (**Table 2**). The results showed that gender, allergic status, CRSwNP, comorbid asthma and serum Af-sIgG were related to disease recurrence in the univariate analysis.

**Figure 3:**
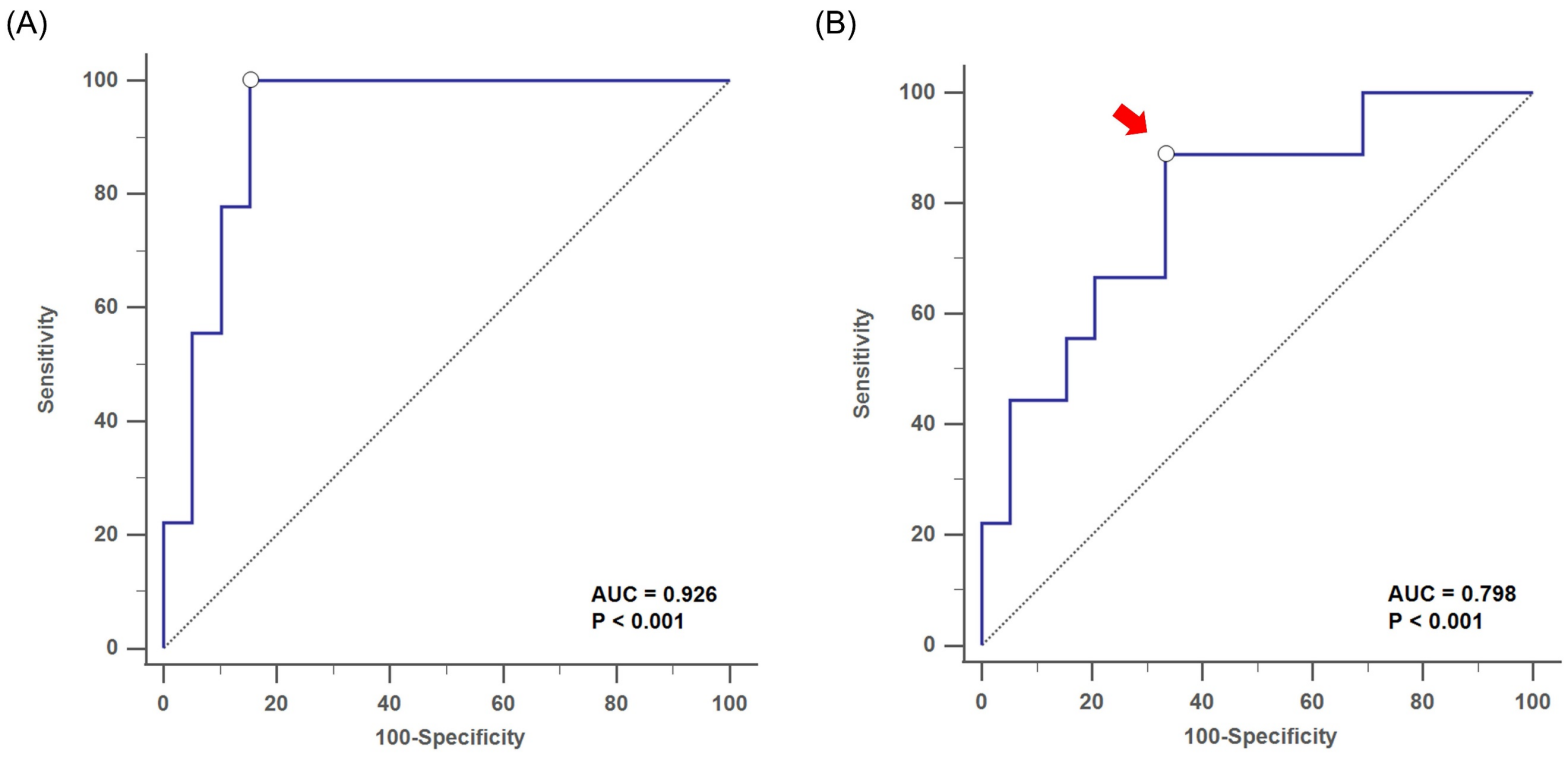
Receiver operating characteristic (ROC) curve analysis showed (A) the discrimination performance of the multivariate logistic regression analysis with the AUC= 0.926, for the evaluation of postoperative recurrence in primary CRS patients (*p* < 0.001). (B) the optimal cutoff value of serum Af-sIgG level (arrow, 27.1 mg/L) to predict postoperative recurrence, with AUC = 0.798 (*p* < 0.001). Abbreviations: CRS, chronic rhinosinusitis; AUC = area under the curve; Af-sIgG, Aspergillus fumigatus-specific immunoglobulin G.

The subsequent MLR analysis showed that the gender (odds: 12.71, 95% CI: 1.2-134.72, *p* = 0.043) and serum Af-sIgG level (odds: 1.06, 95% CI: 1.01-1.13, *p* = 0.034) were independent factors for postoperative recurrence, which implied that the risk of disease recurrence increased by 6% for each 1 mg/L elevation in serum Af-sIgG concentration (**Table 2**). Furthermore, this MLR model showed good discrimination [AUC-ROC = 0.926] and calibration (Hosmer-Lemeshow Ĉ test, *p* = 0.853) (**Figure 3A**).

By using the ROC curve, we obtained an optimal cutoff value of the serum Af-sIgG level to predict postoperative recurrence at 27.1 mg/L, with a sensitivity of 88.9%, a specificity of 66.7%, a PPV of 38.1% and a NPV of 96.3% (area under the curve, AUC = 0.798, *p* < 0.001; **Figure 3B**). Furthermore, logistic regression multivariate analysis showed that a serum Af-sIgG level > 27.1 mg/L was the only significant risk factor for the recurrence of primary CRS, and gender was no longer a significant risk factor in this model. The odds ratio of a higher serum Af-sIgG level was 15.1 (*p* < 0.001 by logistic regression), indicating a 15-fold probability of postoperative recurrence with a preoperative serum Af-sIgG level > 27.1 mg/L (95% CI: 1.26-180.00, *p* = 0.013).

## Discussion

Serum Af-sIgG levels have already been applied in lower airway diseases and have been widely used to help diagnose diseases such as ABPA, make treatment plans, and in some conditions, for disease follow-up 4. In a previous study, there was a significant difference in Af-sIgG levels in acute Af-disease, including ABPA, compared to the control group without acute Af-disease, with a cutoff value of 78.05 mg/L, and the follow-up showed a trend of decreasing Af-sIgG levels after treatment 29. Another study proposed a role of serum Af-sIgG levels in diagnosing ABPA, with a cutoff value of 26.9 ml/L 30. A trend of decreased levels of Af-sIgG was also found after treatment, demonstrating its correlation with disease severity in ABPA 30. The potential relationship between fungal organisms and CRS was first proposed in 1981 31. They presented five patients with CRS, all of whom had pathologic resemblance to the mucoid impaction of ABPA and stated this was the first description of allergic aspergillosis of the paranasal sinuses. However, for decades, no study in the English literature has investigated the role of antibodies against *Aspergillus fumigatus* in primary CRS. In regard to upper airway diseases, Af-sIgG has been mentioned in AFRS and local fungal rhinosinusitis, with Aspergillus colonization in nasal mucosa demonstrated 9. The role of Af-sIgG in common primary CRS remains unmentioned. Accordingly, by the united airway theory, we proposed that fungal colonization in the nasal mucosa may play a role during the inflammatory process in primary CRS.

In this study, we found that patients with T2 CRS and CRSwNP had higher serum Af-sIgG levels than non-T2 CRS and CRSsNP patients, respectively, which indicated that CRS patients with higher serum Af-sIgG levels have more severe disease. By using logistic regression, we obtained an optimal cutoff value for Af-sIgG at 27.6 mg/L to predict the T2 entity for primary CRS. On the other hand, serum Af-sIgG levels were similar in the non-T2 CRS and non-CRS groups. We supposed that serum Af-sIgG levels might be a practical biomarker for the entity classification of primary CRS but not for the existence of CRS.

Furthermore, serum Af-sIgG levels also significantly correlated with the surgical outcome in patients with primary CRS. Those with a serum Af-sIgG level greater than 27.1 mg/L had a 15.1-fold higher risk of postoperative recurrence within one year, and the risk increased by 6% for each 1 mg/L elevation in serum Af-sIgG concentration. These results suggested that the disease endotype, possibility of disease recurrence and magnitude of exposure to the Aspergillus-associated antigen might be connected in primary CRS. The present study may provide physicians with an outcome prediction model before treatment plans are made for primary CRS, and in EPOS 2020, biologics should be taken into consideration as a treatment option for those whose T2 CRS is refractory to standard treatments 5. Accordingly, those with higher serum Af-sIgG levels may be suggested to receive biologics as an alternative or adjuvant treatment, since they may have a higher postoperative recurrence rate. For ABPA, serum Af-sIgG decreased after treatment. However, the data supporting the use of this biomarker for treatment outcome prediction in ABPA is inconsistent 30. Thus, additional studies evaluating the changes in serum Af-sIgG levels after surgical treatment should be conducted in the future.

Limitations of our study include the study population coming from a single institution, a tertiary referral center in northern Taiwan, with possible selection bias in terms of disease severity and territoriality. In addition, limited numbers of patients completed all of the postoperative visits and the lack of a longer follow-up time caused by the pandemic of *coronavirus* disease 2019 (COVID-19) may lead to another form of selection bias. Finally, we had difficulties enrolling healthy volunteers as a control group during the outbreak of COVID-19. A larger study population, a longer follow-up period and normal healthy controls should be enrolled in future studies.

In conclusion, we suggest that the serum Af-sIgG level might be a practical marker to detect T2 inflammation and the surgical outcome in primary CRS. By applying this feasible test, we may be able to achieve optimal treatment for every individual with primary CRS. This study may provide physicians with a reference for future clinical applications in dealing with primary CRS.

## Figures and Tables

**Table 1:** Patient demographics

Variables	T2 CRS (n = 28)	Non-T2 CRS (n = 20)	Non-CRS (n = 22)	*p-value*
Age, years	36.0 (29.0-48.6)	47.5 (34.0-58.7)	37.0 (34.8-44.0)	0.306
Gender, M/F	15/13	9/11	15/7	0.306
Smoking, n (%)	8 (28.6)	6 (30.0)	6 (27.3)	0.622
Allergy, n (%)	20 (71.4)	4 (20.0)	11 (50.0)	0.002*
Asthma, n (%)	5 (17.9)	0 (0)	0 (0)	0.018*
Serum eosinophil count (/uL)	183.7 (80.6-353.6)	95.0 (24.9-127.6)	132.2 (91.0-241.7)	0.036*
Serum total IgE level (KU/L)	146.5 (115.6-259.5)	49.2 (13.5-100.6)	45.7 (32.7-123.2)	< 0.001*
Serum Af-sIgG level (mg/L)	35.9 (22.8-43.7)	19.1 (16.7-22.7)	17.5 (12.1-30.0)	0.012*
LK endoscopy score	5.0 (4.0-6.0)	4.0(4.0-6.0)	N/A	0.741
LM CT score	13.0 (8.0-17.0)	11.0 (8.0-15.7)	N/A	0.470
SNOT-22 score	61.5 (46.0-67.0)	48.0 (33.0-56.9)	40.5 (33.0-55.2)	0.006*

Numerical data: median (95% CI) Categorial data: n (%) *p < 0.05 Abbreviations: T2, type 2; CRS, chronic rhinosinusitis; M, male; F, female; IgE, immunoglobulin E; Af-sIgG, Aspergillus fumigatus-specific immunoglubulin G; LK, Lund-Kennedy; LM, Lund-Mackay; CT, computed tomography; SNOT-22, SinoNasal Outcome Test-22.

**Table 2:** Comparisons between primary CRS patients with and without postoperative recurrence

		Univariate			Multivariate	
Variables	Recurrence (n=9)	Nonrecurrence (n=39)	*p value*	Coefficient	Odds of recurrence	*p value*
Age	36.0 (23.7-57.6)	40.0 (34.0-51.0)	0.475			
Gender, M/F	8/1	16/23	0.023^*^	2.54	12.71 (1.20-134.72)	0.039^*^
Smoking, n (%)	2 (22.2)	6 (15.4)	0.633			
Allergy, n (%)	7 (77.8)	17 (43.6)	0.137	-	-	0.189
T2 CRS, n (%)	7 (77.8)	21 (53.8)	0.204			
Asthma, n (%)	3 (33.3)	1 (2.6)	0.010^*^	-	-	0.202
Nasal polyps, n (%)	8 (88.9%)	25 (64.1%)	0.152	-	-	0.201
Serum eosinophil count (/uL)	229.6 (30.1-460.7)	112.1 (76.2-191.0)	0.391			
Serum total IgE level (KU/L)	125.0 (57.7-145.9)	107.0 (70.7-194.0)	0.892			
Serum Af-sIgG level (mg/L)	40.6 (27.7-69.0)	20.8 (18.6-27.2)	0.006^*^	0.07	1.06 (1.01-1.14)	0.028^*^
LK endoscopy score	4.0 (3.1-6.9)	5.0 (4.0-6.0)	0.548			
LM CT score	13.5 (9.0-16.3)	11.0 (8.0-16.5)	0.391			
SNOT-22 score	65.0 (37.7-77.0)	56.0 (43.9-61.1)	0.328			

Numerical data: median (95% CI) Categorial data: n (%) Odds: value (95% confidence interval, lower-higher) *p < 0.05 Abbreviation: M, male; F, female; CRS, Chronic rhinosinusitis; IgE, immunoglobulin E; Af-sIgG, Aspergillus fumigatus-specific immunoglobulin G; LK, Lund-Kennedy; LM, Lund-Mackay; CT, computed tomography; SNOT-22, SinoNasal Outcome Test-22

## Abbreviations

Af: Aspergillus fumigatus
ABPA: allergic bronchopulmonary aspergillosis
Af-sIgG: Aspergillus fumigatus-specific immunoglobulin G
AFRS: allergic fungal rhinosinusitis
CRS: chronic rhinosinusitis
GMS: Grocott methanamine silver stain
EPOS 2020: European Position Paper on Rhinosinusitis and Nasal Polyps 2020
T2: type 2
FESS: functional endoscopic sinus surgery
CT: computed tomography
CRSwNP: chronic rhinosinusitis with nasal polyps
CRSsNP: chronic rhinosinusitis without polyps
SNOT-22: Sino-Nasal Outcome Test-22
LK: Lund-Kennedy
LM: Lund-Mackay
ROC: receiver operating characteristic
CI: confidence interval
MLR: multivariate logistic regression
PPV: positive predictive value
NPV: negative predictive value
COVID-19: coronavirus disease 2019
